# Retrospective evaluation of the prevalence of known and presumed hereditary eye diseases in a population of Labradoodles referred for ophthalmic screening examinations in the Netherlands

**DOI:** 10.3389/fvets.2026.1841935

**Published:** 2026-06-08

**Authors:** Lauren-Tess Goossens, Anne-Marie J. Verbruggen, Goedele Storms, Bart Broeckx

**Affiliations:** 1 EYES Ophthalmology Clinic for Animals, Breda, Netherlands; 2 Veterinary Practice ‘Kleidal’, Hemiksem, Belgium; 3 Laboratory of Animal Genetics, Department of Veterinary and Biosciences, Faculty of Veterinary Medicine, Ghent University, Merelbeke, Belgium; 4 Centre for Clinical Genetics of Companion Animals, Faculty of Veterinary Medicine, Ghent University, Merelbeke, Belgium

**Keywords:** breeding practices, cataract, designer crossbreed, distichiasis, inherited ocular disorders, persistent pupillary membrane

## Abstract

**Introduction:**

The popularity of designer crossbreed dogs, including Labradoodles, has increased markedly in recent years. This retrospective study evaluated ophthalmic findings and the prevalence of known and presumed hereditary eye diseases (KP-HED) in Labradoodles presented for European College of Veterinary Ophthalmologists (ECVO)-certified screening in the Netherlands.

**Methods:**

ECVO Certificates from Labradoodles examined between January 2014 and December 2024 by a single ECVO diplomate were retrospectively reviewed. Screening consisted of slit-lamp biomicroscopy and indirect ophthalmoscopy following pharmacologic mydriasis. The prevalence of each KP-HED was calculated relative to the total number of dogs examined.

**Results:**

A total of 1,182 Labradoodles (940 females, 242 males; median age 27.8 months, range 4.2–131.7 months) were included. Ninety-two dogs (7.8%) were affected by at least one KP-HED, resulting in 96 diagnoses. The most prevalent conditions were iris-to-iris persistent pupillary membrane (PPM; 4.7%) and distichiasis (2.5%), followed by cataract (0.5%), multifocal retinal dysplasia (MRD; 0.2%), and other lens opacities (0.2%). All cataracts were classified as later-onset and included cortical (0.4%), posterior polar (0.2%), and nuclear (0.08%) subtypes. The most common additional finding was the presence of tiny pigment dots on the central anterior lens capsule (3.9%).

**Discussion:**

Overall, the prevalence of vision-threatening KP-HED in this population was low, indicating a generally favorable ocular health status. However, the relatively higher prevalence of iris-to-iris PPM and distichiasis compared to progenitor breeds suggests a potential influence of genetic background and breeding practices. These findings support the continued use of ophthalmic screening to guide breeding decisions.

## 1 Introduction

In recent years, several countries, including the United States (US), United Kingdom (UK), and the Netherlands have experienced a notable increase in the demand for so-called “designer crossbreed” dogs—purpose-bred crosses derived from two (or occasionally more) distinct purebred progenitor breeds (1–5). A recent UK-based survey found that nearly half (47.1%) of designer crossbreed owners cited hypoallergenicity as a key reason for their choice, a proportion approximately six times higher than that among purebred dog owners (7.86%) (2). Additionally, prospective owners are often motivated by the expectation of better general health and suitability for family life or inexperienced handlers (2). Despite these motivations, scientific evidence supporting reduced hair shedding or lower levels of *Canis familiaris* allergen 1 (Can f 1), a major dog allergen responsible for allergic reactions in humans, in designer crossbreeds is still inconclusive (6, 7).

The Royal Guide Dog Association of Australia is frequently credited with the development of the original designer crossbreed, the Australian Labradoodle, during the 1980s (2, 8). This crossbreed was created by taking the guide dog aptitude of the Labrador Retriever and the low-shedding coat of the poodle into account, with the aim of producing a potentially hypoallergenic working dog (2, 8). To enhance public acceptance, these crossbred puppies were marketed under the hybrid label “Labradoodle” (2, 8). Today, the term “Labradoodle” encompasses both first-generation (F1) hybrids—direct crosses between purebred Labrador Retrievers and poodles—and the Australian Labradoodle, a multigenerational population with genetic contributions from several additional breeds, including the Irish Water Spaniel, Curly-Coated Retriever, and both American and English Cocker Spaniels (9–11). This approach was adopted to develop a consistent breed phenotype through structured crossbreeding. Indeed, a recent genomic admixture study confirmed contributions from several breeds; however, the Labradoodle remains genetically predominantly poodle (11). Allele frequency analysis further demonstrated an enrichment for poodle-associated coat traits, while the contribution of Labrador-specific alleles was minimal (11).

Despite their growing popularity, the classification of the Labradoodle as a crossbreed precludes its recognition by major kennel organizations, including The Kennel Club (UK), the American Kennel Club (US), and the Fédération Cynologique Internationale (FCI) and its affiliated national kennel clubs, such as the Dutch Kennel Club (Raad van Beheer, NL). Nonetheless, dedicated breeder associations exist and impose specific health screening requirements for breeding animals, including eye examinations performed by Diplomates of the European College of Veterinary Ophthalmologists (ECVO), European Eye Scheme Examiners (ESE), or by Diplomates of the American College of Veterinary Ophthalmologists (ACVO) as part of the Orthopedic Foundation for Animals Companion Animal Eye Registry (OFA CAER) program in the United States (12–16). Australian Labradoodles have been presented for ophthalmic examinations under the OFA CAER program since the early 1990s (17). As a result, annual prevalence data for presumed inherited ocular disorders in Labradoodles have been included in the ACVO Genetics Committee Blue Book. However, the cited references and breeding recommendations in the Blue Book pertain exclusively to the Labrador Retriever and poodle parent breeds, rather than being tailored to the Labradoodle population (17).

To date, only a limited number of studies have addressed ophthalmic abnormalities in the Labradoodle population. The most comprehensive study was conducted by *Oliver* and *Gould*, who reported the results of ophthalmic examinations in 435 Labradoodles in the UK (18). In that study, multifocal retinal dysplasia (MRD) was the most common finding with a prevalence of 4.6%, followed by cataract (3.7%), persistent pupillary membrane (PPM) remnants (1.4%), and optic nerve hypoplasia (0.2%). The MRD prevalence was significantly higher than in Labrador Retrievers, while cataract rates were comparable (18).

A large-scale genomic screening study by *Donner* et al. examined the frequency and distribution of 250 genetic disease variants in over 1,000,000 purebred and mixed-breed dogs, including some ophthalmic disorders (19). This study and a previous one by the same author with a smaller cohort revealed that mixed-breed dogs were more likely to carry recessive disease alleles, while purebreds were more often genetically affected (19, 20). This work resulted in the development of *MyBreedData*, an online resource reporting breed-specific carrier and disease prevalence data (20). According to this database, Australian Labradoodles have been identified as heterozygous carriers for progressive rod-cone degeneration (prcd-PRA, NC_006591.3:g.4188663C > T, OMIA variant ID: 69; *PRCD* gene), Stargardt disease (c.4176insC, OMIA variant ID: 1050; *ABCA4* gene), and cone-rod dystrophy 1 (cord1-PRA, OMIA variant ID: 699; *RPGRIP1* gene), a variant first identified in the Miniature Long-Haired Dachshund and subsequently reported in several other breeds (21–24). To date, no genetically affected Labradoodles have been reported in this database. In addition, commercially available genetic screening for Labradoodles is also offered through laboratories such as the University of California, Davis Veterinary Genetics Laboratory, although these datasets are not publicly accessible (25).

A more recent study by *Bryson* et al. collected owner-reported health data for three designer crossbreeds (Cavapoo, Cockapoo, and Labradoodle) and their respective progenitor breeds (Cavalier King Charles Spaniel, cocker spaniel, Labrador Retriever, and poodle) in the UK (26). The odds of the 57 most frequently reported health conditions—including ophthalmic disorders, though not specified individually—were compared between crossbreeds and progenitor breeds. For Labradoodles, no significant differences in the odds of ophthalmic disorders were identified relative to poodles or Labrador Retrievers (26).

Given the current lack of breed-specific data on ocular diseases in Labradoodles, the objective of this study is to report the ophthalmic findings and the prevalence of Known and Presumed Hereditary Eye Diseases (KP-HED) in a population of Labradoodles in the Netherlands presented for ECVO-certified ophthalmic screening examinations.

## 2 Materials and methods

This retrospective study evaluated ECVO Certificates from Labradoodles presented for ECVO-certified ophthalmic screening examinations between January 1, 2014 and December 31, 2024. All dogs were presented for breeding purposes at the initiative of their owners. Both F1 hybrids and Australian Labradoodles of all ages were included in the study upon presentation of an identification document and, when available, previous eye certification, as prescribed by ECVO guidelines (27). Although most Labradoodles were accompanied by breeder-issued pedigree documentation, such documentation was not required for inclusion, as the breed is not officially recognized and the documents therefore lack formal validity. As a result, the term ‘Labradoodle’ throughout this manuscript is used to collectively refer to both F1 hybrids and multigenerational Australian Labradoodles. Each dog included in this study underwent at least one ophthalmic screening examination performed by the same ECVO board-certified veterinary ophthalmologist (AV). The examination included slit-lamp biomicroscopy (SL-15 and SL-17, Kowa Company Ltd.) and binocular indirect ophthalmoscopy (Omega 500, Heine Optotechnik) following pharmacological pupillary dilation with tropicamide 0.5% (Minims Tropicamide, Théa; Aleon Tropicamide; Mydriaticum 0.5%, Théa). Dogs were examined for a number of specific KP-HED as listed on the ECVO Certificate (28). The “descriptive comments” section was used to document any additional findings in the eye and adnexa, either KP-HED or other, using standardized terminology wherever applicable, in accordance with the ECVO Guidelines for the use of the ECVO Certificate (29). For each specified KP-HED listed on the ECVO Certificate, dogs were classified as either “unaffected,” indicating that there was no clinical evidence of the KP-HED specified; “affected,” signifying that there was clinical evidence of the KP-HED specified; or “suspicious/undetermined” (28). The latter classification was used in cases where the animal displayed minor but specific signs of the KP-HED and further development may confirm the diagnosis, or where the animal displayed clinical features that could possibly fit the KP-HED specified, but the changes were inconclusive (28). When at least one specified KP-HED was marked as “suspicious/undetermined,” the owner was advised to present the dog for re-examination within 12 months, in accordance with the ECVO guidelines (29).

### 2.1 Data collection and statistical analysis

Data on sex and age at the time of examination (in months) were extracted from each ECVO Certificate. Dogs presented for multiple screenings were counted once, with only the results from the most recent examination included in the statistical analysis. Bilateral disorders were counted only once, while distinct KP-HED affecting the same eye were counted separately. Descriptive statistics were reported as medians with ranges. The prevalence of each KP-HED was calculated as a proportion of the total number of animals examined. Prevalence analysis was performed as descriptive proportion estimates, and inferential statistics were not applied as the study aimed to describe prevalence rather than test hypotheses. All statistical analyses were performed using R version 4.3.2.

### 2.2 Ethics statement

This retrospective study used existing clinical data and complied with the Guidelines for Ethical Research in Veterinary Ophthalmology (GERVO). No experimental procedures were performed on animals, and ethical review and approval were not required according to local regulations. Written informed consent for the use of clinical data was obtained from all animal owners as part of routine clinical procedures.

## 3 Results

**Figure 1:**
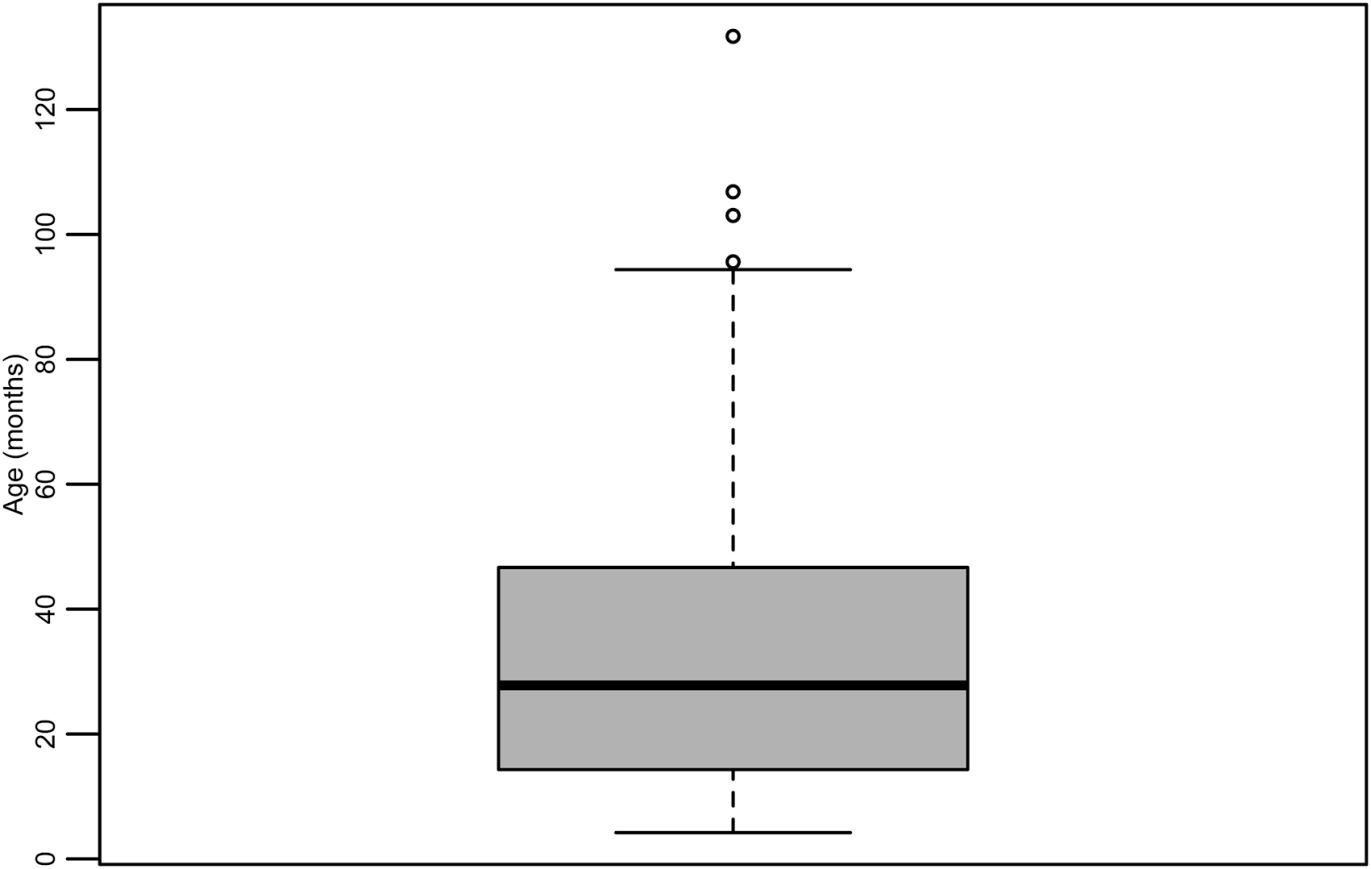
Box plot illustrating the age distribution of the Labradoodles included in the study population (*n* = 1,182). The median age was 27.8 months at the time of examination, with a predominance of young adult dogs and a smaller proportion of older individuals.

A total of 1,182 Labradoodles were examined and included in this retrospective study, comprising 1,041 Australian Labradoodles and 141 F1 hybrids. The study population consisted of 940 intact females and 242 intact males with a median age at the time of examination of 27.8 months (IQR 14.3–46.7; range 4.2–131.7 months; Figure 1). Of the 1,182 dogs, 479 underwent multiple ophthalmic examinations (range 2–5 examinations per dog), resulting in a total of 1915 examination reports. Overall, 92 dogs were diagnosed with at least one KP-HED. Of these, 88 dogs had one KP-HED, while 4 dogs had two KP-HED, resulting in a total of 96 KP-HED diagnoses. The median age of affected dogs was 20.6 months (IQR 13.8–34.8; range 4.6–82.6 months), slightly younger than the overall study population.

### 3.1 Type of known and presumed hereditary eye diseases

**Figure 2:**
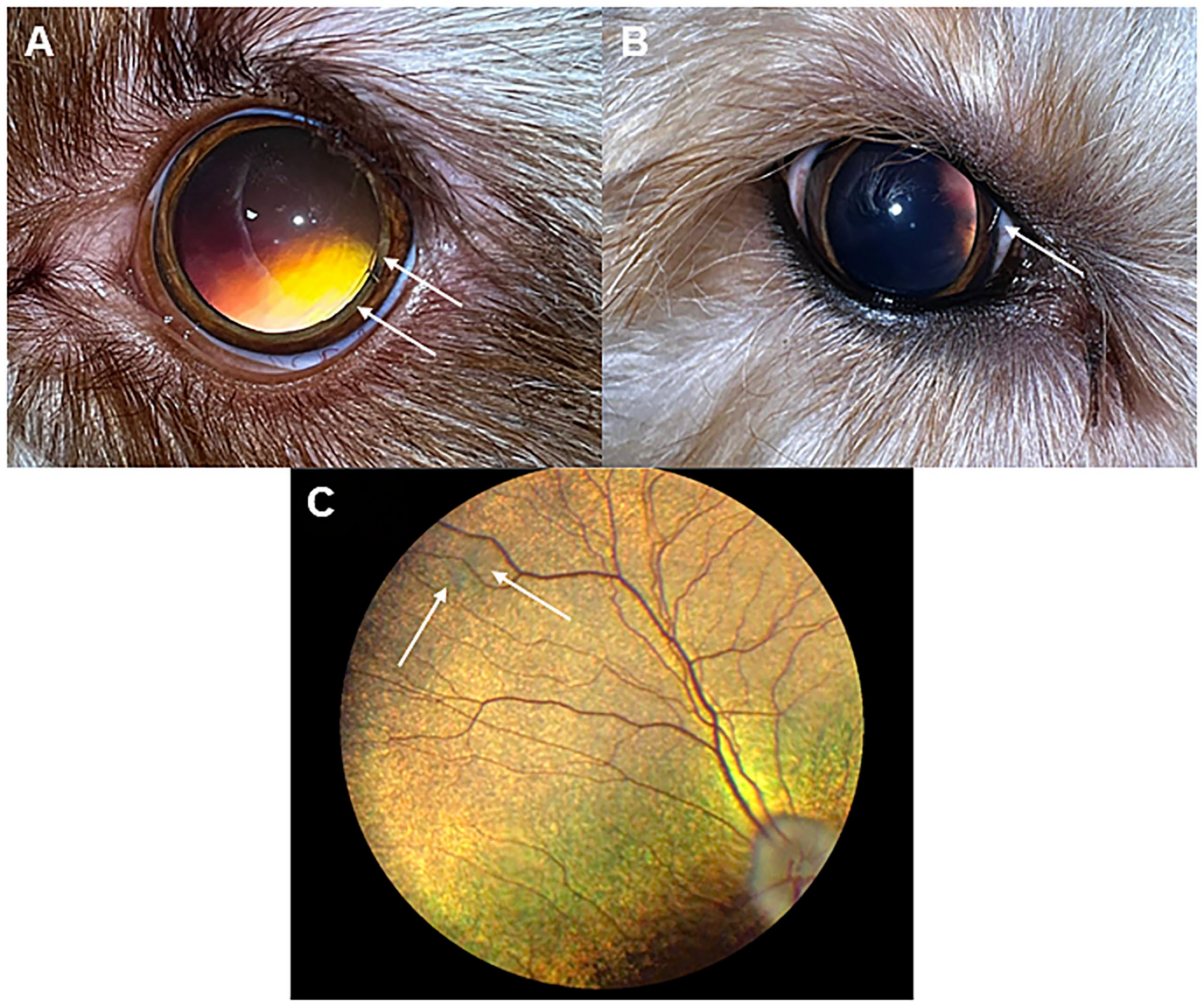
Clinical images of known and presumed hereditary eye diseases (KP-HED) identified during ECVO-certified ophthalmic screening in Labradoodles. **(A)** Iris-to-iris persistent pupillary membrane (PPM) in the left eye of a 3-year-old female Australian Labradoodle. The white arrows indicate a fine iris-to-iris strand extending from the lateral iris collarette and crossing the pupil. **(B)** Distichiasis in the right eye of a 2-year-old female Australian Labradoodle. The white arrow indicates a distichia emerging from the nasal portion of the upper eyelid margin. **(C)** Multifocal retinal dysplasia (MRD) in the left eye of a 1-year-old female Australian Labradoodle. The white arrows indicate a focal retinal fold lesion within the peripheral tapetal fundus.

The type and prevalence of KP-HED identified in this Labradoodle population are summarized in Table 1. The five most common KP-HED were iris-to-iris PPM (*n* = 54, 4.7%; Figure 2), distichiasis (*n* = 30, 2.5%; Figure 2), cataract (*n* = 6, 0.5%), MRD (*n* = 2, 0.2%; Figure 2), and other lens opacities (*n* = 2, 0.2%). All cataracts were classified as later-onset cataracts and were localized as follows: cortical (*n* = 5, 0.4%), posterior polar (*n* = 2, 0.2%), and nuclear (*n* = 1, 0.08%). In two dogs, two types of cataract occurred simultaneously (cortical and posterior polar cataract); therefore, the sum of the subtype prevalences exceeds the overall cataract prevalence.

**Table 1:** Types and prevalence of KP-HED observed in Labradoodles during the study period (2014–2024).

KP-HED	Subtype/specification	Affected dogs (*n*)	Unaffected dogs (*n*)	Undetermined/suspicious (*n*)	Prevalence (%)
Entropion/Trichiasis	–	0	1,181	1	0
Ectropion/Macroblepharon	–	1	1,181	0	0.08
Distichiasis	–	30	1,152	0	2.5
Corneal dystrophy	–	1	1,181	0	0.08
Persistent Pupillary Membrane (PPM)	Iris-to-iris PPM	54	1,128	0	4.7
PHTVL/PHPV	Grade 1	0	1,181	1	0
Cataract	Overall	6	1,168	8	0.5
	Congenital	0	1,181	1	0
	Later onset—cortical	5	1,170	7	0.4
	Later onset—posterior polar	2	1,179	1	0.2
	Later onset—nuclear	1	1,181	0	0.08
Other lens opacity	Overall	2	1,180	0	0.2
	Suture line	1	1,181	0	0.08
	Nuclear ring	1	1,181	0	0.08
Retinal Dysplasia (RD)	Multifocal (MRD)	2	1,177	3	0.2
	Geographical	0	1,181	1	0
PRA	–	0	1,181	1	0
Other: chorioretinopathy	–	0	1,181	1	0

Listed KP-HED are organized in anatomical order from anterior to posterior ocular structures. The total number of cataract-affected dogs (*n* = 6) includes two dogs, each exhibiting both cortical and posterior polar cataract. Consequently, the sum of subtype counts exceeds the total number of affected dogs. KP-HED, known and presumed hereditary eye diseases; PPM, persistent pupillary membrane; PHTVL/PHPV, persistent hyperplastic tunica vasculosa lentis/persistent hyperplastic primary vitreous; RD, retinal dysplasia; MRD, multifocal retinal dysplasia; PRA, progressive retinal atrophy.

Descriptive comments provided in the ECVO Certificates were reviewed and summarized in Table 2. The most commonly reported descriptive comment, tiny pigment dots on the central anterior lens capsule, was noticed in 46 dogs (3.9%). Additional descriptive comments included corneal changes, pigment deposits involving the cornea, iris, and posterior lens capsule, minor lens capsule irregularities, chorioretinal scars, and other incidental ocular alterations. These findings occurred sporadically across different age groups without a clear age-related distribution, as they were recorded only when specifically noted by the examiner. As these findings were not classified as (possible) hereditary ocular diseases, prevalence percentages were not calculated.

**Table 2:** Summary of descriptive comments recorded on ECVO Certificates in Labradoodles during the study period (2014–2024).

Ocular structure	Descriptive comment	Number of dogs (*n*)
Cornea	Perilimbal corneal edema	1
	Corneal lipidosis	1
	Corneal scar with focal cataract due to trauma	1
	Puppy keratopathy	2
Cornea/iris	Corneal and iridal pigmentation at dorsonasal quadrant (to be observed)	1
Lens	Tiny pigment dots on the central anterior lens capsule	46
	Minor whitish irregularity on posterior lens capsule	6
	Minor whitish irregularity on anterior lens capsule	4
	Minor lens imperfection not visible on retroillumination	3
	Tiny pigment dots on posterior lens capsule	2
	Focal cortical cataract due to corneal perforation	1
Vitreous	Vitreous degeneration associated with blunt trauma	1
Retina	Chorioretinal scar/lesion	5
	Pigmented lesion in tapetal fundus (to be observed)	2
	Focal hyperreflection of retinal tapetum (to be observed)	1
	Mild retinal edema dorsal to the optic nerve head	1
Other	Grey “cloud” along the pupil margin at the 3 o’clock position (fibrin/vitreous)	1

Descriptive comments are organized by ocular structure, listed from anterior to posterior. Comments involving both cornea and iris were grouped under a combined “cornea/iris” category. The most commonly reported descriptive comment, tiny pigment dots on the central anterior lens capsule (*n* = 46, 3.9%), is reported with its prevalence in the main text.

**Figure 3:**
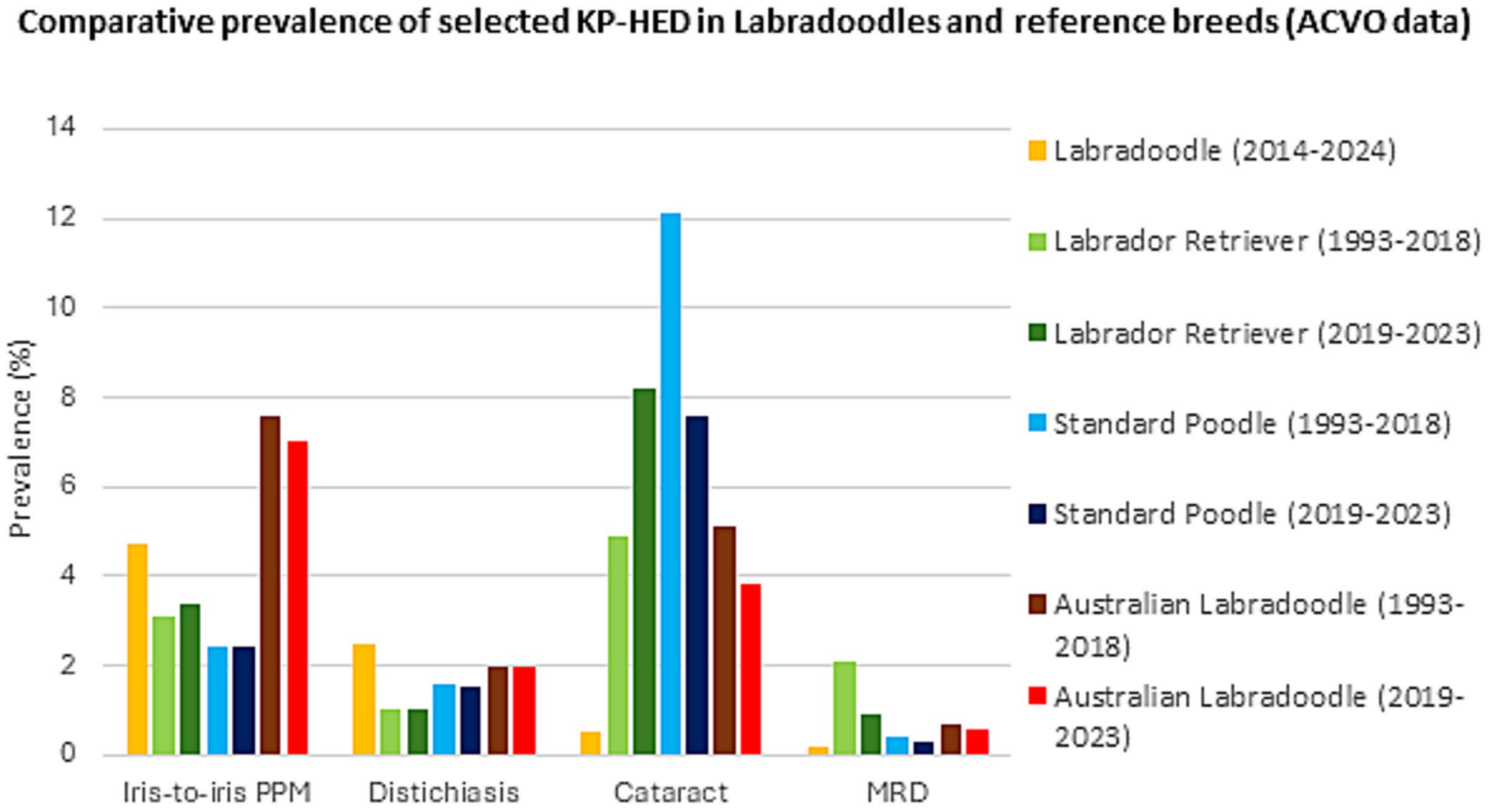
Comparative prevalence (%) of selected KP-HED in Labradoodles from the present study (2014–2024), and in Labrador Retrievers, Standard Poodles, and Australian Labradoodles, based on data reported in the ACVO Genetics Committee Blue Book (17). Data correspond to those summarized in Table 3. KP-HED, known and presumed hereditary eye diseases; ACVO, American College of Veterinary Ophthalmologists; PPM, persistent pupillary membrane; MRD, multifocal retinal dysplasia.

To provide context, the prevalence of selected KP-HED observed in the present study was compared with published prevalence data for the progenitor breeds (i.e., Labrador Retriever and Standard Poodle) and Australian Labradoodle, as reported in the ACVO Genetics Committee Blue Book (Table 3) (17). Labradoodles examined in the present study demonstrated a higher prevalence of iris-to-iris PPM and distichiasis than both parent breeds, but a lower prevalence than Australian Labradoodles. In contrast, the prevalence of cataract and MRD in the present study was markedly lower compared with all three comparison groups. Figure 3 provides a visual overview of these differences to facilitate direct comparison across groups.

**Table 3:** Comparative prevalence (%) of selected KP-HED in Labradoodles from the present study (2014–2024), and in Labrador Retrievers, Standard Poodles, and Australian Labradoodles, based on data reported in the ACVO Genetics Committee Blue Book (17).

KP-HED	Labradoodle^*^ (present study)	Labrador retriever (ACVO)	Standard poodle (ACVO)	Australian labradoodle (ACVO)
Iris-to-iris PPM	4.7	3.1/3.4	2.4/2.4	7.6/7.0
Distichiasis	2.5	1.0/1.0	1.6/1.5	2.0/2.0
Cataract^†^	0.5	4.9/8.2	12.1/7.6	5.1/3.8
MRD	0.2	2.1/0.9	0.4/0.3	0.7/0.6

* The term “Labradoodle” in the present study refers to both F1 hybrids and multigenerational Australian Labradoodles (see Materials and Methods for terminology).

† Cataract prevalence in Labrador Retrievers, Standard Poodles, and Australian Labradoodles includes cases classified as “other lens opacities” in the ACVO Genetics Committee Blue Book. In the present study, these opacities were categorized separately following recently updated ECVO guidelines (29).

For the comparison breeds, two prevalence values are listed per condition, representing the periods 1993–2018 and 2019–2023, respectively. KP-HED, known and presumed hereditary eye diseases; ACVO, American College of Veterinary Ophthalmologists; PPM, persistent pupillary membrane; MRD, multifocal retinal dysplasia; ECVO, European College of Veterinary Ophthalmologists.

## 4 Discussion

This retrospective study of 1,182 Labradoodles presented for ECVO-certified ophthalmic screening in the Netherlands provides insight into the prevalence of KP-HED in this designer crossbreed. The most frequently observed abnormalities were iris-to-iris PPM (4.7%) and distichiasis (2.5%), whereas cataracts were relatively uncommon (0.5%). Other KP-HED occurred at markedly lower frequencies. Overall, the prevalence of vision-threatening hereditary ocular conditions in this screened population was very low.

Compared to the study by *Oliver* and *Gould*, who reported a 4.6% prevalence of MRD and 3.7% prevalence of cataract in 435 UK Labradoodles, a lower prevalence of both conditions was identified in the present study population (0.2 and 0.5%, respectively) (18). In contrast, the prevalence of iris-to-iris PPM was higher in the present study (4.7% vs. 1.4%), and distichiasis was identified in 2.5% of dogs, whereas no data on distichiasis were reported in the UK study. A possible explanation for these discrepancies may involve differences in genetic background and breeding practices between regions. The Australian Labradoodle Association Europe (ALAEU) is headquartered in the Netherlands, and both the Netherlands and Belgium are reported to have the highest density of Australian Labradoodle breeders in Europe (30). It is therefore plausible that the genetic composition of the Dutch Labradoodle population differs from that of the UK population examined by *Oliver* and *Gould*.

Interpretation of prevalence data in Labradoodles should be approached with caution because the breed lacks formal recognition and a standardized definition. As demonstrated by *Ali* et al., the term “Labradoodle” encompasses both F1 hybrids—direct crosses between purebred Labrador Retrievers and poodles—and multigenerational Australian Labradoodles derived from more complex breeding programs involving additional breeds (11). The study by *Oliver* and *Gould* did not specify whether the evaluated dogs were F1 hybrids or multigenerational Australian Labradoodles (18). In the present study, the distinction between F1 hybrids and Australian Labradoodles should likewise be regarded as approximate rather than definitive because of the absence of formal breed recognition. Accordingly, any references to F1 hybrids and Australian Labradoodles in the present study are intended to reflect breeder-reported classification rather than a formally recognized breed status, consistent with current ECVO documentation practices and major Labradoodle breeder association guidelines.

Comparison of the present study population to data reported in the ACVO Genetics Committee Blue Book for the progenitor breeds (i.e., Labrador Retriever and Standard Poodle) as well as the Australian Labradoodle revealed notable differences in the prevalence of selected KP-HED (Table 3) (17). The prevalence of iris-to-iris PPM and distichiasis in the Labradoodles examined in the present study was higher than that reported for Labrador Retrievers and Standard Poodles, whereas it was lower or similar to that observed in Australian Labradoodles. Conversely, the prevalence of cataract and MRD in the present study was markedly lower than in both progenitor breeds and the Australian Labradoodle population. These variations likely reflect differences in genetic background, breeding practices, and population composition, as well as methodological discrepancies between datasets. Specifically, the ACVO Blue Book data originate from a multi-examiner registry system under the OFA–CAER framework in the United States, whereas the present study is based on ECVO-certified examinations performed by a single diplomate within one country. Differences in sampling population, examiner consistency, and screening certification criteria should therefore be taken into account when comparing prevalence estimates. Moreover, the ACVO Blue Book data are derived from substantially larger sample sizes, providing more stable prevalence estimates. Taken together, these factors underscore that inter-program comparisons, while informative, should be interpreted cautiously, as observed differences may primarily reflect methodological variation rather than true disparities in disease prevalence.

Given the composite genetic background of the Labradoodle, it has been hypothesized that this breed could benefit from hybrid vigor or heterosis, a phenomenon in which offspring resulting from the cross of two genetically distinct breeds exhibit improved health, vitality, or other traits relative to the average of their parent breeds (31). However, previous genetic and epidemiological studies have yielded conflicting findings, suggesting that potential hybrid vigor effects may be trait-specific rather than universally beneficial across all inherited disorders. Two large-scale genetic studies conducted by *Donner* et al. demonstrated that, although mixed-breed dogs were more likely to be carriers of common recessive disease variants, purebred dogs were more frequently genetically affected by these conditions (19, 20). According to the online resource *MyBreedData*, based on genotypic data from over 1,000,000 dogs, several heterozygous carriers of progressive rod-cone degeneration (prcd-PRA), Stargardt disease, and cone-rod dystrophy 1 (cord1-PRA) have been identified in the Australian Labradoodle population, whereas no homozygous individuals for these ocular disease variants have been reported in this database (21–24). *Bryson* et al. also investigated the hypothesis of hybrid vigor through a cross-sectional survey comparing health disorders in three designer crossbreeds, including Labradoodles, and their corresponding progenitor breeds (26). The study revealed minimal differences in overall disorder odds, challenging the widespread assumption that designer crossbreeds universally exhibit superior health status. In line with this complexity, prevalence data from the present study and the ACVO Genetics Committee Blue Book further highlight the difficulty of conclusively supporting or refuting hybrid vigor in Labradoodles (17). For example, the prevalence of iris-to-iris PPM and distichiasis in the Labradoodles examined in the present study was higher than what has been reported for Labrador Retrievers and Standard Poodles but lower than for Australian Labradoodles. Conversely, the prevalence of cataract and MRD in the present study was markedly lower than in both progenitor breeds and the Australian Labradoodle population. These mixed prevalence patterns further support the notion that potential hybrid vigor effects may differ between inherited disorders and underscore the complexity of breed health dynamics.

Several limitations should be considered when interpreting the findings in this study. First, the use of a breeding-screening population introduces an important source of selection bias. Dogs with overt ocular clinical signs or advanced ocular disease may be withheld from screening, leading to a possible underestimation of the true prevalence of hereditary eye diseases. However, using a breeding-screening population can also reduce the opposite risk of overestimating prevalence, which is a common issue in clinical populations where only symptomatic animals are presented, thereby exaggerating disease frequency. For example, in a study by *Broeckx* et al. evaluating canine hip dysplasia, the prevalence in a clinical complaint population was over 70%, compared with only 11% in a breeding population and 6% in assistance dogs (32). Similarly, B*eckers* et al. investigated the ABCB1-1Δ mutation, causing multidrug sensitivity in dogs and reported a marked discrepancy in allele frequencies depending on the study population: 21.6% in a cohort of 599 dogs specifically submitted for genetic testing, compared with only 0.2% in a clinical population of 286 dogs presented to a university clinic for routine care (33). These findings highlight how strongly prevalence estimates can depend on study population characteristics and emphasize that the results of this study should be interpreted with caution and may not be extrapolated to the entire Labradoodle population.

In addition, the marked sex distribution imbalance further limits the generalizability of the study population. Approximately 80% of the examined dogs were female, reflecting breeding strategies in which proportionally more females are selected and screened because each male can sire offspring with many females, whereas each female can only produce a limited number of litters during her reproductive lifespan. Consequently, female dogs are more frequently evaluated to ensure suitability for breeding, leading to overrepresentation in screening cohorts. This sex bias has been well documented in genetic studies of purebred dogs, with significantly more reproducing females than males since breed formation, consistent with the popular sire effect (34, 35).

Lastly, the relatively young age distribution of this breeding-screening population limits evaluation of ocular disorders with late onset or slow progression. Although a substantial proportion of dogs (40.5%) underwent multiple ophthalmic screening examinations, the study population still consisted largely of young dogs. Consequently, certain ocular disorders that develop mainly in middle-aged or older dogs may have been underrepresented in the present study. Furthermore, the retrospective design of the study and reliance on ECVO Certificates limited accurate assessment of the exact timing of disease onset and progression.

Another limitation of the present study is the possible underestimation of the historical prevalence of “other lens opacities” and the limited comparability of prevalence data for such opacities across studies and over time. Prior to 2024, these findings were typically documented alongside other types of cataract (e.g., nuclear, cortical, and posterior polar) on the ECVO Certificate and were therefore considered clinically relevant, potentially restricting breeding eligibility. In the updated 2024 ECVO guidelines, however, these opacities are listed separately in the “comment field,” reflecting a shift in their interpretation as clinically less relevant, with breeding now considered optional in affected individuals, which may have contributed to underestimation of their historical prevalence (29). In addition, the ACVO Blue Book applies a different categorization, including “other lens opacities” under cataracts with the exception of Y-suture tip opacities, further complicating direct comparison of prevalence estimates (17) Nevertheless, given that only two dogs in this dataset were classified as having “other lens opacities,” the potential impact on the overall results is very small. Importantly, all examinations in this study were performed by the same ECVO diplomate, ensuring consistency within the dataset and supporting the internal validity of the findings.

A further limitation of the present study is the absence of follow-up data for dogs classified as “suspicious” or “undetermined,” potentially leading to underestimation of the true disease prevalence. These classifications reflected minor or equivocal changes that did not allow a definitive diagnosis at the time of the last examination. Because follow-up examinations were not available within the framework of this retrospective study, it remains unknown whether some of these cases subsequently progressed to clinically apparent disease. However, given that the number of cases classified as “suspicious” or “undetermined” was relatively small, their influence on the overall prevalence estimates might be limited.

Lastly, all examinations were conducted post-mydriasis, potentially limiting detection of certain dynamic ocular abnormalities that may only be apparent without pupil dilation. Nevertheless, all examinations were conducted in accordance with standard ECVO protocols, which highly recommend, but do not mandate, an initial evaluation prior to pupil dilation (29). Furthermore, all examinations in this study were performed by the same ECVO diplomate, minimizing interobserver variability and ensuring consistent application of diagnostic criteria. In conclusion, this retrospective study provides valuable insights into the prevalence of KP-HED in the Dutch Labradoodle population, revealing a generally favorable ocular health status. While certain abnormalities such as iris-to-iris PPM and distichiasis were observed at higher rates compared to the progenitor breeds, the overall prevalence of vision-threatening conditions remained very low. These findings highlight the complexity of inherited ocular disorders in this designer crossbreed, influenced by genetic background and breeding practices. However, the results should be interpreted in light of certain limitations, including the relatively young study population and potential selection bias inherent in screening-based data. Future research incorporating large-scale genomic analyses and longitudinal follow-up studies is warranted to better elucidate hereditary risk factors and to further explore the potential impact of hybrid vigor. Combining ECVO-certified ophthalmic screening data with genetic testing for known disease variants could provide a more comprehensive assessment of hereditary ocular risk in this population and could help validate genotype–phenotype correlations. Moreover, the integration of advanced diagnostic modalities and systematic screening protocols could enhance early detection and inform breeding strategies aimed at maintaining ocular health in Labradoodles over time. In light of these findings, breeders may consider prioritizing screening for PPM and distichiasis in breeding animals, particularly given their relatively higher prevalence. Further refinement of breeding guidelines may help reduce the incidence of these conditions in future generations.

## Data Availability

The raw data supporting the conclusions of this article will be made available by the authors, without undue reservation.
